# Guidelines for antiretroviral therapy in HIV-1 infected adults and adolescents 2014, Thailand

**DOI:** 10.1186/s12981-015-0053-z

**Published:** 2015-04-24

**Authors:** Weerawat Manosuthi, Sumet Ongwandee, Sorakij Bhakeecheep, Manoon Leechawengwongs, Kiat Ruxrungtham, Praphan Phanuphak, Narin Hiransuthikul, Winai Ratanasuwan, Ploenchan Chetchotisakd, Woraphot Tantisiriwat, Sasisopin Kiertiburanakul, Anchalee Avihingsanon, Akechittra Sukkul, Thanomsak Anekthananon, for The Adults and Adolescents Committee of the Thai National HIV Guidelines Working Group

**Affiliations:** Department of Medicine, Bamrasnaradura Infectious Diseases Institute, Ministry of Public Health, Tiwanon Road, Nonthaburi, 11000 Thailand; Bureau of AIDS, TB, and STIs, Department of Disease Control, Ministry of Public Health, Nonthaburi, Thailand; National Health Security Office, Bangkok, Thailand; Thai AIDS Society, Bangkok, Thailand; Department of Medicine, Faculty of Medicine, Chulalongkorn University, Bangkok, Thailand; HIV-NAT, Thai Red Cross AIDS Research Centre, Bangkok, Thailand; Faculty of Medicine Siriraj Hospital, Mahidol University, Bangkok, Thailand; Faculty of Medicine, Khon Kaen University, Khon Kaen, Thailand; Department of Preventive Medicine, Faculty of Medicine Srinakharinwirot University, Nakornnayok, Thailand; Faculty of Medicine Ramathibodi Hospital, Mahidol University, Bangkok, Thailand; Thailand Ministry of Public Health-U.S. CDC Collaboration (TUC), Nonthaburi, Thailand

## Abstract

New evidence has emerged regarding when to commence antiretroviral therapy (ART), optimal treatment regimens, management of HIV co-infection with opportunistic infections, and management of ART failure. The 2014 guidelines were developed by the collaborations of the Department of Disease Control, Ministry of Public Health (MOPH) and the Thai AIDS Society (TAS). One of the major changes in the guidelines included recommending to initiating ART irrespective of CD4 cell count. However, it is with an emphasis that commencing HAART at CD4 cell count above 500 cell/mm^3^ is for public health, in term of preventing HIV transmission and personal benefit. In tuberculosis co-infected patients with CD4 cell counts ≤50 cells/mm^3^ or with CD4 cell counts >50 cells/mm^3^ who have severe clinical disease, ART should be initiated within 2 weeks of starting tuberculosis treatment. The preferred initial ART regimen in treatment naïve patients is efavirenz combined with tenofovir and emtricitabine or lamivudine. Plasma HIV viral load assessment should be done twice a year until achieving undetectable results; and will then be monitored once a year. CD4 cell count should be monitored every 6 months until CD4 cell count ≥350 cells/mm^3^ and with plasma HIV viral load <50 copies/mL; then it should be monitored once a year afterward. HIV drug resistance genotypic test is indicated when plasma HIV viral load >1,000 copies/mL while on ART. Ritonavir-boosted lopinavir or atazanavir in combination with optimized two nucleoside-analogue reverse transcriptase inhibitors is recommended after initial ART regimen failure. Long-term ART-related safety monitoring has also been included in the guidelines.

**Electronic supplementary material:**

The online version of this article (doi:10.1186/s12981-015-0053-z) contains supplementary material, which is available to authorized users.

## Introduction

Combined antiretroviral therapy for the treatment of HIV infection has dramatically improved in both resource-rich and resource-constrained countries. The public health approach to scaling up antiretroviral therapy (ART) in resource-limited situation aims to support the development of treatment programs that can be accessible as widely as possible. Since 2002, the Thai Government Pharmaceutical Organization (GPO), Bangkok, Thailand, has produced GPOvir-S® which is a fixed-dose combination of stavudine (d4T), lamivudine (3TC), and nevirapine (NVP) [1-4]. The Thai GPO has launched many generic antiretroviral drugs afterward, such as tenofovir, efavirenz and lopinavir/ritonavir. Those generic antiretroviral drugs facilitate a drug supply procedure for the national ART program. One of the reasons is to standardize and to simplify treatment regimens and to provide drugs for treatment of drug-resistant viruses. Nowadays, more than 220,000 patients are currently treated with antiretroviral drugs under the support of the National AIDS Program (NAP) and the National Security Program. Non-nucleoside reverse transcriptase inhibitor (NNRTI)-based ART remains to be the first-line recommended regimen for treatment-naive HIV-infected patients in the country to date.

The last version of Thai national guidelines for ART in HIV-1 infected adults and adolescents was published in 2010 [5]. New evidence has emerged regarding when to commence antiretroviral treatment, optimal treatment regimens, the management of HIV co-infection with opportunistic infections, including tuberculosis and others, as well as the management of ART failure. In 2013, WHO has launched and consolidated HIV treatment guidelines by recommending ART for HIV-patients who have CD4 cell count ≤ 500 cells/mm^3^ regardless of WHO clinical stages [6]. Nowadays, the U.S. Department of Health and Human Services (DHHS) panel and the International Antiviral Society-USA panel now recommends that ART should be offered to all HIV-infected adults [7,8]. Such evidences and progresses formed the basis for the new recommendations contained in the 2014 treatment guidelines and were summarized in this publication. The main consideration was based on the risks and benefits of implementing each recommendation, in addition to the acceptability, cost and feasibility. These recommendations aim to provide guidance to HIV-care providers on the appropriate use of antiretroviral drugs for the treatment of HIV infection in adults and adolescents in the country. The key updated consensus recommendations included encouraging earlier HIV treatment irrespective of CD4 cell count and promoting the use of less toxic antiretroviral regimens for the first-line ART, frequency of monitoring HIV treatment response, and drug options for the treatment-experienced patients.

The guidelines were developed by the collaborations of the Department of Disease Control, Ministry of Public Health (MOPH) and the Thai AIDS Society (TAS) The Thai National HIV Guidelines Working Group was appointed to update Thai Guidelines based on their expertise in HIV clinical research, patient care, patient insight, and government policy in Thailand. Relevant published literature and guidelines were reviewed, including clinical studies conducted in Thailand.

### The appropriate timing to initiate treatment

The benefits of ART in decreasing morbidity and mortality in HIV-infected patients with low CD4 cell counts have been well established [9]. The previous national guideline recommended to initiating ART in the patients with a history of an AIDS-defining illness or CD4 cell count <350 cells/mm^3^ [5]. To date, data supporting initiation of ART in patients with higher CD4 cell counts, ranging from 350 cells/mm^3^ to >500 cells/mm^3^, are from large observational studies and randomized controlled trials. There were a number of large cohorts showing that delaying initiation of ART until CD4 count fell below 350 cells/mm^3^ was associated with a greater risk of AIDS-defining illness and/or death than initiating ART at CD4 cell count greater than 350 cells/mm^3^ [10-12]. In addition, SMART, a randomized trial, demonstrated that patients who deferred ART until their CD4 cell counts dropped to <250 cells/mm^3^ had a higher risk of AIDS- and non-AIDS-related events than those who initiated therapy immediately [13]. The study in Thais corresponded with such findings [14]. With regard to patients with CD4 cell counts >500 cells/mm^3^, NA-ACCORD, a large observational cohort study revealed that patients who started ART with CD4 cell counts >500 cells/mm^3^ had a higher adjusted mortality rate than those who did after their CD4 cell counts dropped below this threshold [10]. The evidences thus far showed that earlier ART initiation reduced HIV-related disease progression although the proportion of study among the patients with CD4 > 500 cells/mm^3^ remains less [10,15]. The benefit of ART among patients with high CD4 cell count in reducing transmission of HIV has been shown in the HPTN 052 study [16]. Given that it is placed high value on averting HIV- and AIDS-related death, disease progression and the likely risk of HIV transmission, ART is recommended for all HIV-infected patients regardless of CD4 cell count as shown in Table 1. However, the clinicians should give a priority on the patients with CD4 ≤ 500 cells/mm^3^. For patients who have CD4 > 500 cells/mm^3^, the clinicians should discuss regarding their willingness and commitment to long-term treatment and their understanding the benefits and risks of the therapy and the importance of adherence. With regard to pregnant women, we recommend to initiate ART as soon as possible in all CD4 cell count levels. The goals of treatment are the same with other HIV-infected patients as well as prevention of perinatal transmission of HIV [17,18].

**Table 1:** **Recommendations for antiretroviral therapy initiation in Thai HIV-infected adolescents and adults**

**Recommendations**	**Remarks**
Recommend initiating antiretroviral therapy for all HIV-infected patients regardless of CD4 cell count, especially focus on the patients with CD4 ≤ 500 cells/mm^3^	In case of CD4 > 500 cells/mm^3^, taking into account the following issues
	- Patients have to understand the benefit and side effects of treatment as well as adhering to the regimens
	- Patients may decide to postpone antiretroviral therapy
	- Assess the readiness of the patients to start antiretroviral therapy
	- In case of asymptomatic HIV-infected patients, the major benefit is to decrease rate of HIV transmission

### ART initiation in specific circumstances

#### Tuberculosis co-infection

Remarkable achievements in reducing mortality in HIV and tuberculosis (TB) co-infected patients by ART had been previously reported in Thais [19-22]. The major concern raised is that it needs to balance between mortality associated with delayed ART initiation versus mortality associated with TB-associated immune reconstitution inflammatory syndrome (TB IRIS) and/or severe overlapping drug hypersensitivity/toxicities with early ART. Over the past few years, six randomized trials, including one study in Thailand, had addressed how early ART should be initiated [19,23-27]. Most of the studies showed a lower mortality rate in HIV and TB co-infected patients, particularly in patients with CD4 < 50 cells/mm^3^. One recent study of those with high CD4 cell count showed no difference between early and late ART on composite endpoint of death, tuberculosis treatment failure, and recurrent rate of TB [26]. It is recommended that all HIV-infected patients with active TB should be treated with ART. In patients with CD4 counts <50 cells/mm^3^ and those with CD4 counts ≥50 cells/mm^3^ who have severe clinical disease, ART should be initiated within 2 weeks of starting TB treatment as shown in Table 2. With regard to the patients with TB meningitis, however there was a higher rate of severe adverse events without clinical benefit of early ART initiation [25]. Thus, initiation of ART is at 2 weeks after TB treatment is recommended.

**Table 2:** **Recommendations for antiretroviral therapy initiation in Thai HIV-infected adolescents and adults with active major opportunistic infections**

**Opportunistic infections**	**≤ 50 cells/mm** ^**3**^	**> 50 cells/mm** ^**3**^	
		More severe*	**Less severe**
Tuberculosis	Within 2 weeks	Within 2 weeks	Between 2–8 weeks
Cryptococcosis	Between 4–6 weeks		
Pneumocystis pneumonia	Between 2–4 weeks		
Mycobacterium avium complex infection			
Others			
Cytomegalovirus infection	As soon as possible		
Progressive multifocal leukoencephalopathy			
Cryptosporidium infection			

*More severe clinical disease was defined as low Karnofsky score, low body mass index [BMI], low hemoglobin, low albumin, organ system dysfunction, or extent of disease.

#### Cryptococcosis co-infection

Although survival benefit from ART was achieved in Thai patients co-infected HIV and cryptococcosis [28-30], optimal timing for ART initiation in patients with acute cryptococcal meningitis remains unclear. The previous published reports showed inconsistent outcomes [31,32]. A systematic review compared the clinical and immunologic outcomes for early initiation ART (less than four weeks after starting antifungal treatment) versus later initiation of ART (four weeks or more after starting antifungal treatment) in HIV-positive patients with concurrent cryptococcal meningitis [33]. There was insufficient evidence in support of either early or late initiation of ART to date. Nevertheless, it is recommended that ART initiation should be delayed until 4–6 weeks after initiation of fungal treatment as shown in Table 2. Previous studies showed that 8%–50% of AIDS patients with cryptococcosis developed cryptococcal IRIS after initiation of ART [34-36]. Therefore, IRIS should be vigorously managed if ART is initiated earlier.

### *Pneumocystis* pneumonitis

With regard to *Pneumocystis* pneumonitis, a randomized control trial demonstrated lower rate of mortality and disease progression in the patients who received early ART at a median of 12 days after treatment of opportunistic infection. All most one-third of the patients enrolled into this study were diagnosed *Pneumocystis* pneumonitis [31]. However, severe IRIS has been reported [37]. Thus, all patients should be carefully monitored after the first few weeks of ART initiation. It is recommended to start ART between 2–4 weeks after starting treatment for *Pneumocystis* pneumonitis. Recommendations for ART initiation in patients with *Pneumocystis* pneumonitis and other opportunistic infections are shown in Table 2. All HIV-infected patients with moderate-to-severe *Pneumocystis* pneumonitis, defined by room air pO_2_ < 70 mm Hg or alveolar-arterial O_2_ gradient ≥35 mm Hg, should receive adjunctive corticosteroids combined with specific therapy [38].

### Recommended initial ART regimens in antiretroviral naïve HIV-infected patients

Optimal initial ART regimens for antiretroviral naïve HIV-infected patients should consist of two nucleoside reverse-transcriptase inhibitors (NRTIs) plus the third antiretrovirals. The NNRTIs are potent but have the limitation of a low genetic barrier to drug resistance that requires only a single mutation to confer high level of drug resistance except etravirine [39]. Nonetheless, due to its proven long-term efficacy in various large scale trials, its availability, less drug-drug interaction and its low cost [40], the guidelines recommend to use non-nucleoside reverse-transcriptase inhibitor (NNRTI) as the third agent in combination to 2 NRTIs. A fixed-dose combination is preferred. The current guidelines still emphasized on avoidance of d4T as a preferred option because of its risk of mitochondrial toxicity [41], and should consider switching to another NRTI in currently treated patients.

Efavirenz-based ART has been extensively used worldwide for more than a decade and it has shown durability in treatment-naive patients [40,42-45]. This regimen has comparable or superior virologic responses to all current available drugs in the country and this drug showed consistent virologic responses across any plasma HIV levels and CD4 counts [46]. In addition, efavirenz-based ART regimen can be given once daily and is co-formulated with tenofovir and emtricitabine. Thus, efavirenz is recommended as the third drug combined with NRTI backbone. If the patients cannot tolerate efavirenz due to neuropsychiatric adverse events and others, the next NNRTI option is either rilpivirine or nevirapine. The pooled results of ECHO and THRIVE demonstrated that the proportion of patients with viral suppression at 48 weeks was comparable in the rilpivirine and efavirenz-containing arms [47]. Rilpivirine was well tolerated. However, a higher rate of HIV resistance-associated mutations among patients with a baseline HIV viral load >100,000 copies/mL, the risk of virologic failure was higher in the rilpivirine arm compared with the efavirenz arm [48,49]. Plasma HIV RNA should be measured prior to starting rilpivirine and this drug is not recommended in the patients with baseline HIV viral load >100,000 copies/mL. In addition, patients should be well informed on 2 limitations of rilpivirine i.e., should be taken with food to optimize its absorption and should be aware of some relevant drug-drug interactions. There were no significant differences in terms of virologic or immunologic outcomes between treatment-naive patients assigned to nevirapine or efavirenz-based ART in the 2NN study [42]. However, a recent systematic review showed that efavirenz-based ART is significantly less likely to lead to virologic failure compared to nevirapine-based ART [50]. Nevirapine is more toxic during the first three months of therapy [42]. Therefore, nevirapine has been changed from recommended NNRTI to be an alternative to efavirenz. A dose of nevirapine 200 mg once daily is recommended for the first 14 days prior to increasing to usual dose at 200 mg twice daily. However, nevirapine should not be initiated in women with a baseline CD4 cell count >250 cells/mm^3^ or in men with a CD4 cell count >400 cells/mm^3^ because these CD4 thresholds represent major risk factors for liver toxicity. If the patients cannot tolerate all three recommended NNRTIs, the next alternative third agents are protease inhibitors, including lopinavir/ritonavir or atazanavir/ritonavir.

With regard to NRTI backbone, virologic responses were significantly higher among patients receiving tenofovir/emtricitabine compared with zidovudine/lamivudine [51]. Tenofovir in combination with emtricitabine or lamivudine is recommended as the preferred backbone. Because tenofovir, emtricitabine and lamivudine have activities against both HIV and hepatitis B virus, thus tenofovir plus emtricitabine or lamivudine is also recommended in the patients with such co-infection [52,53]. If tenofovir is contraindicated, the alternative NRTIs include abacavir or zidovudine. Stavudine is no longer recommended as first-line drug due to its high rate of toxicities as aforementioned. The patients who have received stavudine, regardless the development of its adverse events, should switch to tenofovir, while their plasma HIV viral load is still undetectable. Currently available antiretroviral drugs in Thailand and the recommended dosages are summarized in Table 3. Initial and alternative ART regimens in antiretroviral naïve HIV-infected patients are summarized in Table 4. In terms of antiretroviral-naïve pregnant women, more data regarding safety of efavirenz are available and it provides increased reassurance for recommending this drug [54]. Thus, efavirenz combined with tenofovir plus emtricitabine or lamivudine is recommended as a first-line therapy.

**Table 3:** **Current available antiretroviral drugs in Thailand**

**Generic Name**	**Abbreviation**	**Dosage form**	**Adult dose**
**Nucleoside reverse transcriptase inhibitors (NRTIs)**			
Lamivudine	3TC	150, 300 mg, 10 mg/ml	150 mg q 12 h or 300 mg OD
Abacavir	ABV	300 mg	300 mg q 12 h or 600 mg OD
Zidovudine	AZT	100, 300 mg, 10 mg/ml	200 - 300 mg q 12 h
Stavudine	d4T	15, 20, 30 mg	30 mg q 12 h
		5 mg/ml	
Didanosine	DDI	250, 400 mg (extended release capsule)	≤60 kg 250 mg OD, > 60 kg 400 mg OD
		25, 125, 200 mg (Buffer tablet)	with TDF ≤60 kg 200 mg OD, > 60 kg 250 mg OD on empty stomach
Tenofovir	TDF	300 mg	1 tab OD
**Combined NRTIs**			
Zidovudine + Lamivudine	AZT/3TC	300/150 mg	1 tab q 12 h
Abacavir + Lamivudine	ABV/3TC	600/300 mg	1 tab OD
Tenofovir + Emtricitabine	TDF/FTC	300/200 mg	1 tab OD
Stavudine + Lamivudine	D4T/3TC	30/150 mg	1 tab q 12 h
**Non-Nucleoside reverse transcriptase inhibitors (NNRTIs)**			
Rilpivirine	RPV	25 mg	1 tab OD with meal
Efavirenz	EFV	50, 200, 600 mg	600 mg OD hs, on empty stomach to reduce side effect
Etravirine	ETR	100 mg	2 tab q 12 h with meal
Nevirapine	NVP	200 mg, 10 mg/ml	200 mg q 12 h or 400 mg OD
**Combine NRTIs + NNRTIs**			
Tenofovir + Emtricitabine + Efavirenz	TDF/FTC/EFV	300/200/600 mg	1 tab OD hs, on empty stomach to reduce side effect
Stavudine + Lamivudine + Nevirapine	GPO vir S	30/150/200 mg	1 tab q 12 h
Zidovudine + Lamivudine + Nevirapine	GPO vir Z 250	250/150/200 mg	1 tab q 12 h
**Protease inhibitors (PIs)**			
Atazanavir	ATV	200, 300 mg	300 mg OD (boosted rtv), 400 mg OD, with TDF 300 mg OD (boosted rtv) with EFV
			400 mg (boosted rtv), with meal
Darunavir	DRV	300, 600 mg	600 mg q 12 h, 800 mg OD with meal
Indinavir	IDV	200, 400 mg	800 mg q 12 h
Lopinavir/ritonavir	LPV/rtv	100/25, 200/50 mg, 80/20 mg/ml	400/100 mg q 12 h or 800/200 mg OD
Ritonavir	RTV	100 mg	boosted RTV 100 mg q12 h or OD
Saquinavir	SQV	500 mg	2 tab q 12 h
**CCR5 Inhibitor (CCR5I)**			
Maraviroc	MRV	150, 300 mg	150 mg q 12 h with strong cyp 3A inh. (with or without 3A ind.) ex. PIs except TPV/r
			300 mg q 12 h with NRTIs, T20, NVP, RAL,TPV/r and other drug that not strong 3A inh./ind.
			600 mg q 12 h with cyp 3A ind ex. EFV, ETR (without 3A inh.)
**Integrase inhibitors**			
Raltegravir 400 mg	RAL	400 mg	1 tab q 12 h

**Table 4:** **Initial and alternative antiretroviral therapy regimens in antiretroviral naïve HIV-infected patients**

**NRTI backbones**		**NNRTIs**		**Other third drugs**
**Recommended drugs**		**Recommended drug**		**Recommended drug**
Tenofovir/emtricitabine	plus	Efavirenz	In case the patients cannot tolerate NNRTIs	Lopinavir/ritonavir
Tenofovir/lamivudine				Atazanavir/ritonavir
**Alternative drugs**		**Alternative drugs**		
Abacavir + lamivudine		Rilpivirine		
Zidovudine + lamivudine		Nevirapine		

NRTI = Nucleoside reverse transcriptase inhibitor.

NNRTIs = Non-nucleoside reverse transcriptase inhibitors.

### Antiretroviral therapy in treatment-naive patients who co-infected with HIV and tuberculosis

Treatment with antiretroviral drugs in patients who co-infected with HIV and tuberculosis is relatively complex. These issues include poor tolerability of concomitant treatment regimens, drug co-toxicities, polypharmacy impacts on adherence, as well as pharmacokinetic drug interactions between rifampicin and antiretroviral drugs [55,56]. A rifampicin-based anti-tuberculosis regimen is essential [57]. All patients with HIV-related tuberculosis should be treated with a rifampicin-containing regimen for the full course of tuberculosis treatment [57]. Rifampicin induces hepatic cytochrome P-450 resulting in a significant decrease of plasma NNRTI and protease inhibitor concentrations [58,59]. For patients who are receiving non-rifampicin containing anti-tuberculosis regimens, the recommended ART regimens are the same as for non-tuberculosis patients. For patients who are treated with rifampicin-containing anti-tuberculosis regimen, efavirenz-based ART is recommended. The dosage of efavirenz is no longer needed adjustment by weight. Standard dose of efavirenz was efficacious in the patients who were receiving rifampicin in Thais [60,61]. Thus, all patients are recommended to receive the dosage of efavirenz at 600 mg/day. A number of studies in Thailand have shown that nevirapine at a normal dose of 400 mg/day can be used effectively with rifampicin [59,62,63]. One study found that an increase of nevirapine dose to 600 mg per day, with a lead-in of 200 mg twice daily, was associated with a high rate of liver toxicity and is therefore not recommended [62]. Thus, a standard dose of nevirapine is an alternative to efavirenz for patients treated with rifampicin, and lead-in nevirapine treatment (200 mg/day) during the first 2 weeks is not necessary [64]. Rilpivirine is contraindicated in patients taking rifampicin. In case the patient cannot tolerate efavirenz- or nevirapine- containing ART, the other alternative drug is raltegravir at 400 mg twice daily [65]. There is limited clinical experience with use of concomitant raltegravir and rifampicin, especially in larger and long-term trials. Thus, this regimen should be prescribed with caution.

### Laboratory monitoring of antiretroviral therapy

Recommendations on the CD4 cell count, plasma HIV viral load monitoring, and other relevant laboratory tests are summarized in Table 5. HIV viral load and CD4 cell count are key surrogate markers of HIV treatment response before and after ART initiation, respectively [7]. Decreases in plasma HIV viral load following ART initiation are associated with reduced risk of progression to AIDS or death. This guideline recommends to monitor plasma HIV viral load regularly at 3 months and 6 months in the first year of ART and at least yearly afterward. With regard to immunologic response, CD4 cell count should be monitored at 6 months and 12 months in the first year and until CD4 cell count >350 cells/mm^3^ and viral load <50 copies/mL. HIV genotypic drug-resistance testing is recommended to guide treatment choices after virological failure. This test should be performed in case of plasma HIV viral load >1,000 copies/mL and it should be performed while the patient is taking antiretroviral drugs or within 1 month after discontinuing ART.

**Table 5:** **Recommendations on the CD4 cell count, HIV viral monitoring, and other tests**

**Laboratory testing**	**At entry into care**	**First year of ART**	**After first year**
CD4 cell count	At entry	At 6 and 12 months	At 6 and 12 months until CD4 count > 350 cells/mm^3^ with viral load <50 copies/ml and once a year afterward.
Plasma HIV viral load	-	At 3 and 6 months	Every 12 months if viral load <50 copies/ml
HBsAg	At entry	-	-
Anti-HCV	At entry	-	-
VDRL	At entry	-	-
ALT	At entry	If indicated	At entry
Creatinine	At entry	At 6 and 12 months	Every 12 months or if indicated
Total cholesterol	High risk group	Every 12 months or if indicated	Every 12 months or if indicated
Fasting blood sugar	High risk group	Every 12 months or if indicated	Every 12 months or if indicated
Urinalysis	At entry	Every 12 months or if indicated	Every 12 months or if indicated

### Antiretroviral therapy in treatment-experienced patients

New drugs that have new mechanisms of action and activity against drug-resistant viruses have been added in the guidelines. Based on current available drugs in the country, the ultimate goal to treat treatment-experienced patients is to re-achieve virologic suppression to <50 copies/mL [66]. Thus, a careful review of the previous antiretroviral history, all previous HIV-1 genotypic resistance test results, potential drug-drug interactions, relevant co-morbidities, antiretroviral drug availability, and a patient’s adherence is important to properly plan for selecting the next ART regimen.

### First ART regimen failure

#### NNRTI-based first-line failure

The SECOND-LINE and EARNEST studies recently showed that second-line regimens consisting of a boosted-protease inhibitor plus 2 NRTIs/NtRTIs and boosted-protease inhibitor plus raltegravir led to favorable treatment outcomes for patients with HIV after initial NNRTI-based regimen failure [67,68]. In protease inhibitor-naive patients failing NNRTI-based first-line ART, mono-therapy with ritonavir-boosted lopinavir had a significantly lower proportion of patients with undetectable viral load compared to the ritonavir-boosted lopinavir plus tenofovir and lamivudine [69]. Thus, ritonavir-boosted lopinavir monotherapy is not recommended as a second-line option. Antiretroviral treatment options after first-line NNRTI-based regimens are summarized in Table 6.

**Table 6:** **Antiretroviral options after first-line non-nucleoside reverse transcriptase inhibitor-based regimens**

**NRTI in failing regimen**	**NRTI option in next regimen**	**Third agent**
Tenofovir	Choosing NRTI based on genotypic resistant result or considering zidovudine plus lamivudine	Recommended agent: lopinavir/ritonavir
		Alternative agents: Atazanavir/ritonavir
		Darunavir/ritonavir
		Raltegravir*
		Dolutegravir
Zidovudine, stavudine or abacavir	Choosing NRTI based on genotypic resistant result or considering tenofovir plus lamivudine or emtricitabine	

*Used with caution in non-fully active NRTI backbone owing to rapid emerging of treatment failure.

NRTI = Nucleoside reverse transcriptase inhibitor.

#### Boosted-protease inhibitor based first-line failure

In patients failing an initial protease inhibitor-based regimen, NRTI resistance mutations are commonly observed and protease inhibitor resistance-associated mutations are rare. Nevertheless, protease inhibitor resistance-associated mutations are accumulated in patients with late detection of virologic failure. Choosing a new protease inhibitor in the new regimen is based on the number and patterns of protease inhibitor resistance mutations [39]. An active protease inhibitor, boosted with ritonavir, should be used as a second regimen combined with the two other active drugs, either NRTI or NNRTI, as indicated by the genotypic resistance test results.

#### Multiclass ARV failure

In case of multi-class failure, the subsequent regimen should be consisted of new three fully active drugs, including integrase inhibitors (raltegravir or dolutegravir), protease inhibitors (daurunavir/ritonavir), NNRTIs (etravirine or rilpivirine), and CCR5 inhibitors (maraviroc) based on the genotypic resistance test results. If this is not possible, at least two active drugs are required or expert consultation is advised. The challenge is, however, not all listed new ARVs are available and accessible in Thailand.

## Conclusions

The 2014 HIV treatment guidelines provide the key updated data regarding when to commence antiretroviral treatment, optimal treatment regimens, the management of HIV co-infection with opportunistic infections, treatment monitoring, as well as the management of ART failure. However, the guidelines are not able to provide guidance on care to cover all patients’ circumstances. Thus clinicians should make proper decision on the basis of their patient circumstances. Of note, the success of life-long antiretroviral therapy is critically depended on the attitude, the knowledge, the skill of the health care providers, but more important the commitment of the patients through the good relationship and support from the health care providers.
